# Generating Multi‐Depth 3D Holograms Using a Fully Convolutional Neural Network

**DOI:** 10.1002/advs.202308886

**Published:** 2024-05-09

**Authors:** Xingpeng Yan, Xinlei Liu, Jiaqi Li, Yanan Zhang, Hebin Chang, Tao Jing, Hairong Hu, Qiang Qu, Xi Wang, Xiaoyu Jiang

**Affiliations:** ^1^ Department of Information Communication Army Academy of Armored Forces Beijing 100072 China; ^2^ National Digital Switching System Engineering and Technological Research Center Zhengzhou 450001 China; ^3^ Information Engineering University Zhengzhou 450001 China

**Keywords:** computer‐generated hologram, fully convolutional neural network, multi‐depth hologram

## Abstract

Efficiently generating 3D holograms is one of the most challenging research topics in the field of holography. This work introduces a method for generating multi‐depth phase‐only holograms using a fully convolutional neural network (FCN). The method primarily involves a forward–backward‐diffraction framework to compute multi‐depth diffraction fields, along with a layer‐by‐layer replacement method (L^2^RM) to handle occlusion relationships. The diffraction fields computed by the former are fed into the carefully designed FCN, which leverages its powerful non‐linear fitting capability to generate multi‐depth holograms of 3D scenes. The latter can smooth the boundaries of different layers in scene reconstruction by complementing information of occluded objects, thus enhancing the reconstruction quality of holograms. The proposed method can generate a multi‐depth 3D hologram with a PSNR of 31.8 dB in just 90 ms for a resolution of 2160 × 3840 on the NVIDIA Tesla A100 40G tensor core GPU. Additionally, numerical and experimental results indicate that the generated holograms accurately reconstruct clear 3D scenes with correct occlusion relationships and provide excellent depth focusing.

## Graphical abstract

This work introduces the forward–backward‐diffraction framework for computing multi‐depth diffraction fields and the layer‐by‐layer replacement method for handling occlusion relationships. When combined with a fully convolutional neural network, it generates multi‐depth holograms with excellent depth focusing and corrects occlusion relationships. The reconstructed scene exhibits minimal speckle noise and few edge artifacts.

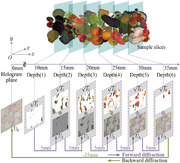

## 1 Introduction

Compared to other 3D display technologies, such as head‐mounted displays,^[^ ^1^ ^]^ autostereoscopic displays,^[^ ^2^ ^]^ and volumetric displays,^[^ ^3^ ^]^ holography^[^ ^4^, ^5^ ^]^ is considered the most promising technology for realizing true‐to‐life 3D displays.^[^ ^6^, ^7^, ^8^ ^]^ It has the ability to encode both the amplitude and phase of a 3D scene into an interference pattern. Through diffraction, it can faithfully reconstruct the original scene, providing all‐natural visual depth cues required by the human vision system,^[^ ^9^, ^10^ ^]^ including occlusion, eye accommodation, (con‐)vergence, and stereopsis. However, due to limitations of experimental setup complexity and performance flexibility, it has been challenging to reconstruct large and complex 3D scenes, especially for virtual 3D scenes, with optical holography. In recent years, with the development of computer technology,^[^ ^11^ ^]^ researchers can obtain the light field information of any 3D scene using graphic computing platforms and generate the corresponding holograms computationally.^[^ ^12^, ^13^ ^]^ This technology is called computer‐generated hologram (CGH) and combines traditional holography with computer technology. According to the types of information contained in CGH, CGH can be divided into phase‐only CGH, amplitude‐only CGH, and complex‐amplitude CGH.^[^ ^10^, ^14^, ^15^ ^]^ The phase‐only CGH imitates the propagation of light emitted from an object scene, usually without the need for reference light, directly converting complex amplitude information in the diffracted field into phase information. Examples include Wirtinger hologram (WH) based on iterative thinking^[^ ^16^ ^]^ and double‐phase hologram (DPH) utilizing heuristic coding approximation.^[^ ^17^ ^]^ Due to its higher diffraction efficiency and broader applicability,^[^ ^18^, ^19^ ^]^ phase‐only CGH has been selected as the primary focus of our study in this paper. Henceforth, the term “CGH” mentioned in the subsequent sections specifically refers to phase‐only CGH. CGHs can be employed to generate the hologram for some large, outdoor, moving, or virtual scenes, and exhibits tremendous flexibility. CGHs can be discretized and printed on an optically sensitive medium,^[^ ^20^, ^21^ ^]^ encoded by a meta surface material,^[^ ^22^, ^23^ ^]^ or loaded onto the core component called the “spatial light modulator (SLM),” or even other novel optical modulator such as anisotropic leaky‐mode modulator,^[^ ^24^ ^]^ to achieve a refreshable and dynamic 3D display.^[^ ^25^, ^26^, ^27^ ^]^ The reconstructed 3D scene can be observed by the researchers at a designed location along the optical path. In addition to the 3D display, CGHs are also used in other fields such as interferometry,^[^ ^28^, ^29^ ^]^ beam shaping,^[^ ^30^, ^31^ ^]^ optoelectronic computing,^[^ ^32^, ^33^ ^]^ optical encryption,^[^ ^34^, ^35^ ^]^ and angular momentum holography.^[^ ^36^, ^37^ ^]^

According to the basic element representation used in CGHs, several kinds of algorithms have been developed to generate CGHs, such as point cloud,^[^ ^38^ ^]^ lines,^[^ ^39^ ^]^ curves,^[^ ^40^ ^]^ polygons,^[^ ^41^ ^]^ layers,^[^ ^42^ ^]^ ray‐tracing,^[^ ^43^ ^]^ and so on. However, the nature of computer computation causes CGHs to suffer from a compromise between reconstructing a more realistic scene and achieving greater computational efficiency. It has often been referred to as the “computational bottleneck.”^[^ ^44^ ^]^ The non‐local many‐to‐many mapping of the diffraction integral gives rise to tremendous computation along with massive memory access and update, which becomes more serious when the resolution of the CGH increases. Meanwhile, accurately modeling light propagation requires a high‐bit depth complex‐valued representation, which is extremely time‐consuming. The additional complex amplitude encoding will also require extra time if the CGH is loaded on a phased‐modulated SLM. Some acceleration methods have also been developed to speed up the calculation of CGH, such as the look‐up table,^[^ ^45^ ^]^ wavefront‐recording‐plane method,^[^ ^43^ ^]^ stereogram approximation,^[^ ^46^ ^]^ Fourier domain sparsity,^[^ ^47^ ^]^ and wavelet transform.^[^ ^48^ ^]^ Besides the algorithm development, the hardware acceleration has also been carried forward, such as CPU,^[^ ^49^ ^]^ GPU,^[^ ^49^ ^]^ and FPGA.^[^ ^11^ ^]^

The generation of the CGH can be viewed as an inverse problem. Optimization algorithms, such as iterative phase‐retrieval algorithms^[^ ^50^, ^51^, ^52^ ^]^ or (stochastic) gradient descent,^[^ ^13^, ^16^ ^]^ are employed to solve this problem. These algorithms iteratively seek a phase‐only pattern that produces a propagated wavefront matching the desired amplitude distribution. Recently, as a prosperously developed tool for optimization, a machine learning approach called deep neural network has been employed as a new paradigm for solving inverse problems in various fields of imaging, photonics,^[^ ^53^, ^54^, ^55^ ^]^ and holography.^[^ ^56^, ^57^ ^]^ In CGH, the DNN is used to solve the complex non‐linear optimization process. The differentiable nature of wave propagation and the maturity of differentiable software infrastructures have nurtured learning‐based CGH algorithms that address the high computational cost.^[^ ^56^ ^]^ The DNN also takes the advantage of the flexibility of modifying the network architecture, tuning network parameters, improving the training algorithms, as well as optimizing the hardware systems. Deep learning techniques have been employed to generate high‐quality holographic images and a multi‐level loss function has been proposed to optimize the training process of wave propagation models.^[^ ^58^ ^]^

Compared to planar holograms, 3D holograms require focusing at different depths, making them more computationally demanding and harder to generate. Traditional methods for 3D CGH generation involve complex algorithms and significant computational resources. In contrast, the DeepCGH approach leverages the power of deep learning to simplify and optimize the hologram generation process.^[^ ^59^ ^]^ Furthermore, a novel deep neural network architecture and training strategy for multi‐depth hologram generation is presented.^[^ ^60^ ^]^ It can advance holography techniques by harnessing the power of deep learning, thereby opening up new possibilities for realistic and efficient 3D imaging applications. And some holograms can be synthesized by optimizing a wave field to reconstruct multiple varifocal images.^[^ ^61^ ^]^ They are rendered through a physically‐based renderer, providing real‐world‐like defocus blur and achieving photorealistic reconstruction. A large‐scale CGH dataset with 4000 pairs of RGB‐depth images and corresponding 3D holograms is designed to train a deep‐learning‐based CGH pipeline, which can generate photorealistic color 3D holograms from a single RGB‐depth image in real time.^[^ ^5^ ^]^

Multi‐depth holograms have the ability to reconstruct 3D scenes focused at different depth planes and demonstrate excellent defocusing blur when the depth information does not match the reconstruction distance. The generation of holograms poses an ill‐posed inverse problem,^[^ ^62^, ^63^ ^]^ while the generation of multi‐depth holograms presents an even greater level of challenge. Despite attempts to improve them, some methods like stochastic gradient descent and Gerchberg–Saxton which are commonly used for generating 2D holograms prove to be mediocre when applied to multi‐depth holograms, resulting in unsatisfactory outcomes.^[^ ^64^, ^65^ ^]^ Furthermore, as a result of inadequate network architecture and improper handling of occlusion relationships, some deep learning‐based methods yield subpar scene clarity and fall short of achieving accurate occlusion culling in the reconstructed scenes.^[^ ^60^, ^66^ ^]^

Here, we propose a forward–backward‐diffraction framework for the first time to compute multi‐depth diffraction fields and combine it with a carefully designed fully convolutional neural network (FCN) to generate multi‐depth holograms, to the best of our knowledge. This framework implements occlusion of the foreground on the background's diffraction field during the forward propagation process of the diffraction field and determines the reconstruction distance through the backward propagation of the diffraction field. In addition, we utilize 3D modeling software to acquire 3D graphical datasets and introduce a multi‐depth loss function to improve the fitting performance of the FCN. During the reconstruction process of a 3D scene, the absence of information from occluded objects leads to prominent dark boundaries between different layers, thereby compromising the visual continuity of the 3D scene. To address this issue, we propose the layer‐by‐layer replacement method (L^2^RM) to smooth the boundaries between different layers and handle the occlusion relationships among them. Finally, we validate the effectiveness of the generated multi‐depth holograms through numerical and optical experiments, showcasing their high reconstruction quality and excellent defocus blur.

## 2 Computation of Multi‐Depth Diffraction Field

Designing a 3D scene and obtaining its multi‐depth diffraction field are essential prerequisites for generating multi‐depth holograms. This section outlines the acquisition of 3D graphical datasets using modeling software and proposes a forward–backward‐diffraction framework to compute its multi‐depth diffraction field.

### 2.1 3D Graphical Datasets

In the process of generating a 3D CGH with neural networks, high‐quality 3D graphical training datasets are often scarce. In traditional methods, researchers typically use depth cameras to record real 3D scenes for obtaining training datasets.^[^ ^67^ ^]^ However, the traditional method is costly, and the operation is quite complicated. For example, time‐of‐flight depth cameras^[^ ^68^, ^69^ ^]^ consist of components such as light sources, optical elements, sensors, control circuitry, and processing circuitry. These cameras are known for their high cost and using them for sampling 3D scenes can be easily affected by multiple reflections.

**Figure 1:**
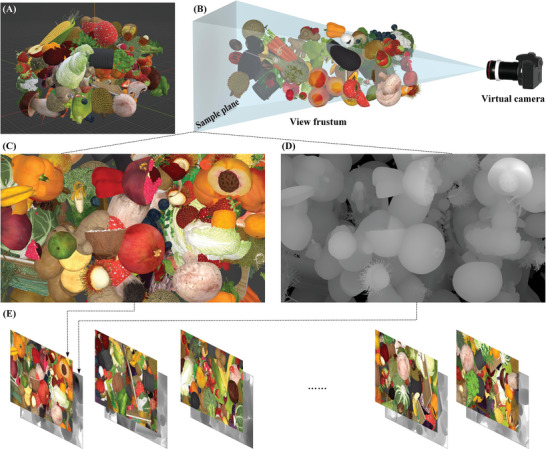
The generation of 3D graphical dataset. A) 3D random scene. B) Sampling process. C) Intensity image. D) Depth image. E) 3D graphical dataset.

A 3D modeling software named “Blender” is used to flexibly and conveniently sample 3D scenes for generating 3D graphical datasets. Compared to real cameras, using virtual cameras for sampling does not cause barrel distortion or pincushion distortion in the images. As shown in **Figure** **1**A, various models of fruits and vegetables are combined randomly into a 3D scene. In this 3D scene, each model has a random size, location, and rotation angle. A virtual camera is employed to capture the 3D scene to get the intensity‐ and depth‐image, which is used to make up the 3D dataset, as shown in Figure 1B. The resolution of the training dataset is set to 2160 × 3840 during the sampling process. We positioned the virtual camera far away from the 3D scene and set its focal length to 350 mm. This helps to avoid any distortion in model size caused by perspective. Before each sample, the 3D scene will be updated with a new random combination of models. Thus, each intensity image and its corresponding depth image in the training dataset will be unique, as shown in Figure 1C,D. Their values are distributed within the range of 0–1. We sampled 900 randomly generated 3D scenes and assigned 800 sets of depth images as the training dataset, while 100 sets of depth images were designated as the test dataset.

### 2.2 Forward–Backward‐Diffraction Framework

**Figure 2:**
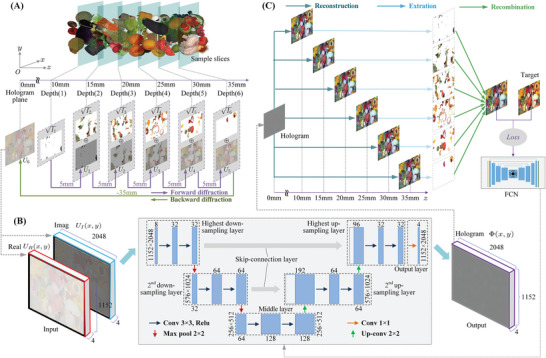
The generation of multi‐depth holograms by FCN. A) The computation of the multi‐depth diffraction field using the forward‐backward‐diffraction framework. B) The structure of the FCN. C) The calculation of multi‐depth error. The complex amplitude $U_{n}\left(i=1,2,\text{…},6\right)$ and the wave field $\sqrt{I_{n}}\left(i=1,2,\text{…},6\right)$ in (A) are depicted as an image for simplicity, even though they actually consist of real and imaginary parts actually.

The calculation process of the multi‐depth diffraction field is demonstrated using **Figure** **2**A as an example. The intensity information *I* of the 3D scene is divided into six sample slices as shown in Figure 2A, which have the same depth interval of Δ*z* = 5mm. According to the dissemination sequence of the diffraction field in the reconstruction process, these slices are respectively located at the depth planes (*i*) of *z_i_* = 10 + (*i* − 1) × 5mm (*i* = 1, 2, ⋅⋅⋅, 6), and the hologram plane is located at *z*_0_ = 0mm. We set the initial phase of slices to 0. The sample slice's wave filed $\sqrt{I_{1}}$ in the depth plane (1) propagates forward to the depth plane (2), and the first diffraction field *U*_1_ is obtained. The superposition of the sample slice's wave filed $\sqrt{I_{2}}$ in the depth plane (2) and the first diffraction field *U*_1_ propagate forward to the depth plane (3), and the second diffraction field *U*_2_ is obtained. By analogy, the superposition of the sample slice's wave filed $\sqrt{I_{5}}$ in the depth plane (5) and the fourth diffraction field *U*_4_ propagate forward to the depth plane (6), and the fifth diffraction field *U*_5_ is obtained. Finally, the superposition of the sample slice's wave filed $\sqrt{I_{6}}$ in the depth plane (6) and the fifth diffraction field *U*_5_ propagate backward to the hologram plane, and the sixth diffraction field (the multi‐depth diffraction field) *U*_6_ is obtained. It should be noted that in the process of forward propagation of the diffraction field, directly adding the sample slice to the previous diffraction field is inaccurate. Instead, a mask is needed to remove the light field corresponding to the sample slice in the diffraction field before adding the sample slice. This is done to simulate the occlusion effect that occurs when the light field of a 3D scene propagates in reality. In the paper, we use the symbol “⊕” to represent this operation method.

Without loss of generality, the above process can be extended to the general situation. Suppose the scenes are divided into *N* sample slices, according to the angular spectrum theory^[^ ^70^ ^]^ the complex amplitude of the *n* ^th^ diffraction field *U_n_* (*x*,*y*) can be expressed using the angular spectrum method (ASM) as

(1) $$\begin{array}{c} U_{n}\left(x,y\right)=\left\{\begin{array}{c} F^{-1}\left\{F\left\{\sqrt{I_{n}\left(x,y\right)}⊕U_{n-1}\left(x,y\right)\right\}\cdot H_{\Delta z}\left(f_{x},f_{y}\right)\right\},1\leq n<N\\ F^{-1}\left\{F\left\{\sqrt{I_{N}\left(x,y\right)}⊕U_{N-1}\left(x,y\right)\right\}\cdot H_{-z_{N}}\left(f_{x},f_{y}\right)\right\},n=N \end{array}\right. \end{array}$$

where *F*{·} is the 2D Fourier transform and *F* ^−1^{·} is the 2D Fourier inverse transform. *U*_0_(*x*,*y*) = 0. *I_n_* (*x*,*y*) is the intensity distribution of the scene in the *n* ^th^ sample slice. The operation method "⊕" is expressed as

(2) $$\sqrt{I_{n}\left(x,y\right)}⊕U_{n-1}\left(x,y\right)=\sqrt{I_{n}\left(x,y\right)}+U_{n-1}\left(x,y\right)\cdot M_{n}\left(x,y\right)$$

where *M_n_* (*x*,*y*) is the mask corresponding to the *n* ^th^ sample slice. *d*(*x*, *y*) is the discrete depth distribution of the 3D scene.

(3) $$M_{n}\left(x,y\right)=\left\{\begin{array}{c} 1,d\left(x,y\right)\neq \frac{z_{n}-z_{\min}}{z_{\max}-z_{\min}}\\ 0,d\left(x,y\right)=\frac{z_{n}-z_{\min}}{z_{\max}-z_{\min}} \end{array}\right.$$

This method for handling occlusion relationships will be discussed in detailed in Section 4.1. *f_x_* and *f_y_* are respectively the spatial frequency of the diffraction field in x‐axis and y‐axis. *H_z_* (*f_x_*,*f_y_*) is the optical transfer function (OTF) in the propagation process of the diffraction field and expressed as

(4) $$H_{z}\left(f_{x},f_{y}\right)=\exp\left[jkz\sqrt{1-{\left(\lambda f_{x}\right)}^{2}-{\left(\lambda f_{y}\right)}^{2}}\right]$$

where j is the imaginary unit, j^2^ = −1. λ is the wavelength of the laser used and k=2π/λ. $\left|f_{x}\right|,\left|f_{y}\right|<1/2\Delta p$ and Δ*p* is the pixel interval of the SLM.

As described above, we refer to this diffraction field propagation model as the “forward–backward‐diffraction framework,” which initially utilizes forward propagation to address the occlusion relationship between the layer and the diffraction field, and then employs backward propagation to determine the scene's diffraction distance. In contrast to the traditional multi‐depth method based on a series of backward diffractions, this diffraction computation framework can handle foreground occlusion on the background's diffraction field, thereby eliminating excess diffraction fields and avoiding unwanted plane wave components interfering with the reconstructed scene. This “forward diffraction + backward diffraction” working mode can both correctly handle occlusion relationships and achieve accurate depth focusing. Ultimately, through the coordination of this forward–backward‐diffraction framework and occlusion processing, a multi‐depth diffraction field *U_N_* (*x*,*y*) of the 3D scene at the plane where the SLM is positioned is obtained.

## 3 Generation and Optimization of Multi‐Depth Holograms

After the multi‐depth diffraction field of the 3D scene's dataset is prepared, the construction of a fully convolutional neural network is required to generate multi‐depth holograms. Furthermore, it is crucial to calculate the multi‐depth errors between the reconstructed images at various diffraction distances from the hologram and the target scene, aiming to train the network for enhancing the reconstruction quality of the holograms.

### 3.1 Network Structure

A U‐Net‐based FCN is designed to generate multi‐depth holograms in this work. The deep convolutional layers of U‐Net allow it to effectively learn intricate features and preserve fine details from diffraction fields. With its ability to capture both local and global context, U‐Net generates high‐quality and realistic holographic outputs while utilizing limited training data. The details of the FCN are shown in Figure 2B. The multi‐depth diffraction field *U_N_* (*x*,*y*) is decomposed into real part *U_R_* (*x*,*y*) and imaginary part *U_I_* (*x*,*y*), and then they are as the input of the FCN, while the phase‐only hologram Φ(*x*, *y*) is output. To reduce the edge fringes of the diffraction fields resulted from the diffraction, the resolution is expanded from 2160 × 3840 to 2304 × 4096 by zero padding. The “phase recombination” method is used to optimize the structure of the input and output layers to reduce the memory footprint and promote generation speed,^[^ ^71^ ^]^ thus the 2304 × 4096×2 input information is converted to a matrix that is 1152 × 2048 × 8 in size, and the input layer of the network has eight channels. In the processing of input information, multiple 3 × 3 × 8 convolutional kernels perform convolutions on the input data with a stride of one. This operation can restructure the real and imaginary parts and link them together. In addition to the input layer, the FCN includes two down‐sampling layers, two up‐sampling layers, a middle layer, an output layer, and a skip‐connection layer.

As mentioned above, the process of generating holograms from the FCN involves two down‐sampling and two up‐sampling steps. In the first down‐sampling step, the feature of the highest down‐sampling layer is extracted from input information using two 3 × 3 convolutional operations and two ReLU functions. This feature becomes the input of the second down‐sampling layer after a 2 × 2 max‐pooling operation. The feature at the second down‐sampling layer is obtained in a similar way to the first and serves as the input to the middle layer. After a 2 × 2 up‐convolution operation, the second up‐sampling layer takes the feature extracted from the middle layer as input. Similarly, the highest up‐sampling layer takes the feature extracted from the second layer as input. Finally, the output of the FCN is obtained from the highest up‐sampling layer feature through a 1 × 1 convolution operation.

In general, down‐sampling is intended to compress the input information, while up‐sampling is designed to restore it. During down‐sampling, the resolution of the input information will be reduced, while the number of its channels will be increased. Conversely, the resolution of the input information will be increased, and the number of its channels will be decreased during up‐sampling. The feature extracted during down‐sampling is concatenated with the feature extracted during up‐sampling using skip‐connections in order to retain more high‐resolution details contained in high‐layer features. The multi‐depth hologram Φ(*x*, *y*) can be obtained from the output of FCN through “phase recombination.”

### 3.2 Training Methods

The designed FCN implements the coding operation $\left[U_{R}\left(x,y\right),U_{I}\left(x,y\right)\right]\overset{\mathrm{FCN}}{\to }\Phi \left(x,y\right)$ in a complex and implicit way. However, before training the network model, the relationship between the input and output is unclear, and the holograms' reconstructed 3D scenes have poor performance. Therefore, the errors between the target and reconstructed scenes need to be well‐defined to update the network's node parameters, and the back‐propagation algorithm is employed. When generating 2D scene holograms using neural networks, only the error of one depth plane needs to be calculated. However, the multi‐depth hologram can reconstruct 3D scenes on different depth planes, so it is necessary to calculate the multi‐depth errors for different depth planes respectively. An L1 (average absolute error) loss function is adopted to calculate the multi‐depth error between the reconstructed scenes and the target scenes, which is defined as:

(5) $$Loss=L_{1}\left\{\sqrt{I\left(x,y\right)},\frac{\sum \sum \left|U^{\prime }\left(x,y\right)\right|\sqrt{I\left(x,y\right)}}{\sum \sum {\left|U^{\prime }\left(x,y\right)\right|}^{2}}\left|U^{\prime }\left(x,y\right)\right|\right\}$$

where *I*(*x*, *y*) is the overall intensity distribution of the 3D scene and *U*′(*x*, *y*) is the recombination of the reconstructed diffraction field. Equation (5) deviates from commonly used normalization methods by aligning $\sqrt{I(x,y)}$ and *U*′(*x*, *y*) in the energy domain. This approach effectively reduces the brightness disparity caused by high‐value noise pixels, thereby preventing loss oscillation and facilitating network convergence.

(6) $$U^{\prime }\left(x,y\right)=\sum_{n=1}^{N}{U^{\prime }}_{n}\left(x,y\right)\left[1-M_{n}\left(x,y\right)\right]$$

*U*′_*n*_(*x*,*y*) is the complex amplitude of the reconstructed diffraction field at the *n* ^th^ depth plane by the multi‐hologram Φ(*x*, *y*), which is expressed as

(7) $${U^{\prime }}_{n}\left(x,y\right)=F^{-1}\left\{F\left\{\exp\left[j\Phi \left(x,y\right)\right]\right\}\cdot H_{z_{n}}\left(f_{x},f_{y}\right)\right\}$$

Figure 2C shows the above calculation process of the multi‐depth error. After obtaining the reconstructions of the multi‐depth hologram at all depths using ASM, the focused parts from each reconstruction are extracted and recombined into a reconstructed scene. Finally, the loss between this reconstructed scene and the target scene is calculated to update the parameters of the network model.

## 4 Enhancement of Occlusion Effects

Occlusion is a critical visual cue in 3D displays, which means that the light field of an object located far away from the observer will be occluded by other objects that are closer to the observer as the light propagates forward. If the issue of occlusion is not effectively addressed, it will lead to a deterioration in the reconstruction quality of the 3D scene.

### 4.1 Standard Method

**Figure 3:**
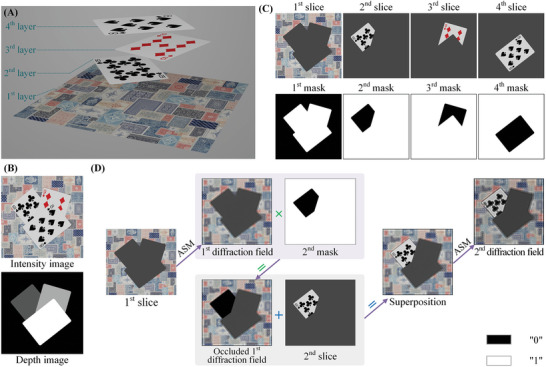
Occlusion processing. A) The 3D scene featuring “pokers.” B) The sampling results. C) The sample slices and “0‐1″ masks. D) Processing occlusion relationships with the standard method.

As shown in Figure 2A, the forward–backward‐diffraction framework presented in Section. 2 attains occlusion effects through the application of a mask for hard‐blocking the diffraction field. In this paper, we refer to this conventional approach to occlusion handling as the “standard method.” Additionally, we have designed a 3D scene featuring “pokers” with four layers to provide a detailed illustration of the standard method. As illustrated in **Figure** **3**A, this 3D scene comprises four layers, sequentially arranged from the first to the fourth layer as follows: the tablecloth, the 10 of clubs, the 9 of diamonds, and the 8 of spades. Figure 3B presents the sampling results of the 3D scene, featuring both the intensity and depth images. In the depth image, the depth of each layer is proportional to the gray value. Figure 3C shows the sample slices and their corresponding “0‐1” masks, which are calculated from Equation (3). In the *n* ^th^ mask, “0” means that the *n* ^th^ slice occludes the (*n* − 1)^th^ diffraction field at the corresponding position, while “1” means that the *n* ^th^ slice doesn't occlude the (*n* − 1)^th^ diffraction field at the corresponding position.

The standard method for handling occlusion relationships implemented via Equation (2) is shown in Figure 3D. First, compute the diffraction field following the propagation of the first slice to depth plane (2) using ASM. As this diffraction field results from the propagation of the first slice, it is referred to as the first diffraction field. Subsequently, multiply the first diffraction field with the second mask derived from the second slice to obtain the occluded first diffraction field, in order to achieve the occlusion effect of the second slice on the first slice. After achieving the occlusion effect, superimpose the occluded first diffraction field and the second slice to propagate toward the depth plane (3) to obtain the second diffraction field. Subsequent steps follow a similar pattern, such as multiplying the second diffraction field with the third mask derived from the third slice to achieve the occlusion effect of the third slice on the first and second slices. Then, continue propagating the superposition of the occluded second diffraction field and the third slice and so forth.

### 4.2 Layer‐by‐Layer Replacement Method

**Figure 4:**
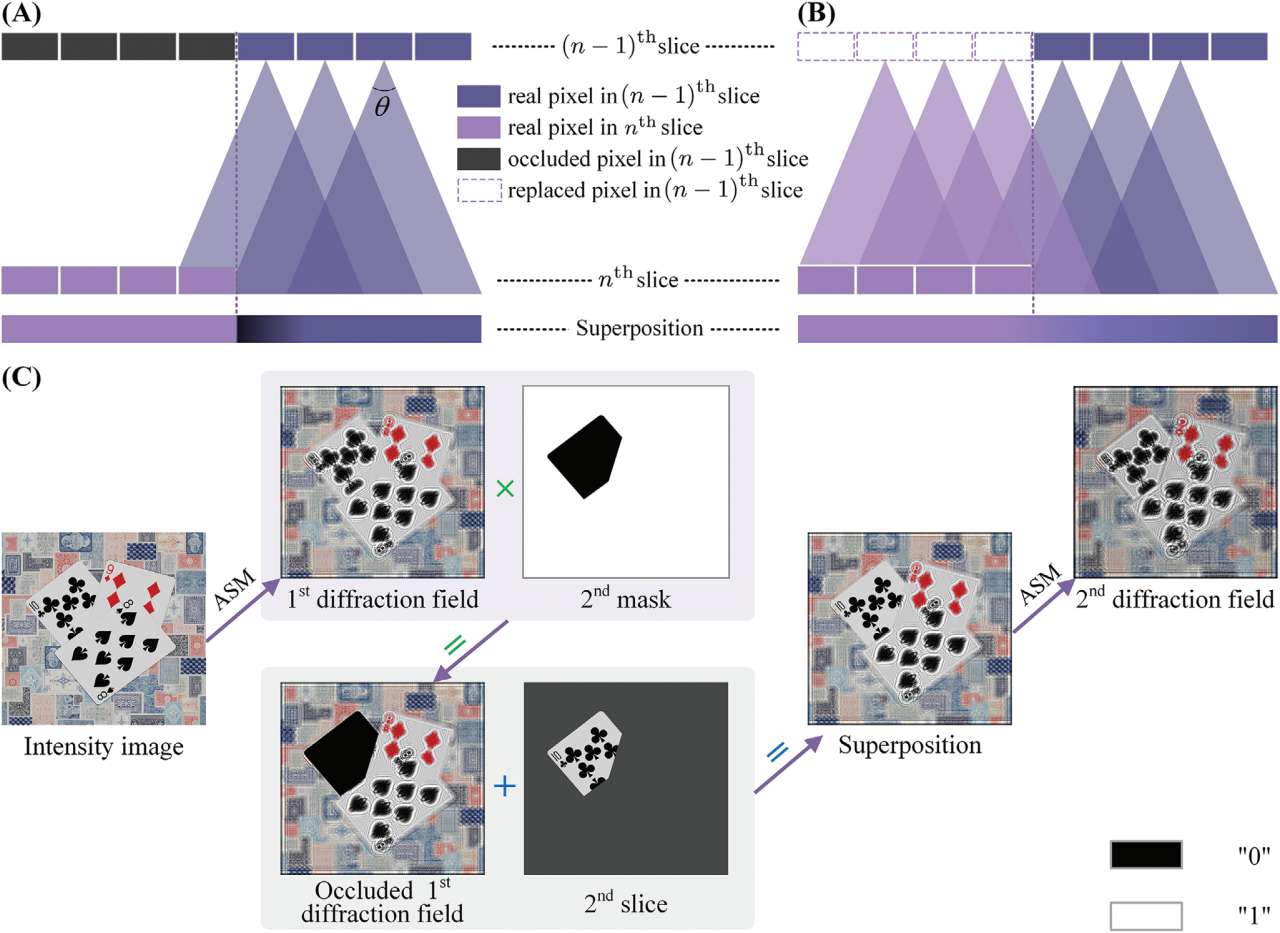
Occlusion enhancement. Analysis of A) the standard method and B) L^2^RM. C) Enhancing occlusion effects with L^2^RM.

While employing the standard method to process occlusion relationships, the absence of information regarding the occluded objects can result in the appearance of distinct dark fringes at the boundaries of different layers, a phenomenon known as the “diffraction missing phenomenon,” clearly visible in Figure 6. This phenomenon is related to the initial phase of the slices. When calculating the diffraction field of the slices, a random distribution of initial phases leads to severe speckle noise in the diffraction field^[^ ^72^ ^]^; therefore, we set the initial phase of the slices to 0. However, waves with the same initial phase will produce coherent superposition, resulting in fringes at the edges of the diffraction field. The absence of information from occluded objects causes the boundaries between slices to be equivalent to the edges of the light field, leading to the appearance of similar fringes at corresponding positions in the diffraction field. The dark line closely adjacent to the boundaries of different slices in Figure 6 represents the most prominent dark fringe in these patterns. They disrupt visual continuity and reduce the reconstruction quality of the 3D scene. **Figure** **4**A depicts the disruption of visual continuity caused by this dark fringe, where the dark purple rectangles represent real pixels of the (*n* − 1)^th^ sample slice and the bright purple rectangles represent real pixels of the *n* ^th^ sample slice.

In this paper, the layer‐by‐layer replacement method (L^2^RM) is proposed as a means to mitigate the presence of the diffraction missing phenomenon in multi‐depth diffraction fields and the reconstructed scenes of multi‐depth holograms. As shown in Figure 4B, the first step involves replacing the occluded pixels in the (*n* − 1)^th^ sample slice with known pixels at the corresponding position in the *n* ^th^ sample slice or in a higher‐level sample slice. Next, we compute the diffraction field of the (*n* − 1)^th^ sample slice and once again replace the pixels in the (*n* − 1)^th^ diffraction field with those at the corresponding position of the *n* ^th^ layer. Due to the filling of missing pixels, the boundaries of the computed diffraction field will become smoother and can be seen as the continuation of the *n* ^th^ sample slice.

The process of utilizing L^2^RM to enhance occlusion effects is shown in Figure 4(C). The intensity image propagates forward, resulting in the generation of the first diffraction field. Subsequently, the second mask occludes the first diffraction field, allowing for the superposition of the latter with the second sample slice to propagate forward together, yielding the second diffraction field. By analogy, each superposition of the diffraction field and the sample slice is replaced by the next sample slice at the corresponding position after diffraction. This calculation process can be expressed as

(8) $$\begin{array}{c} U_{n}\left(x,y\right)=\left\{\begin{array}{c} F^{-1}\left\{F\left\{\sqrt{I\left(x,y\right)}\right\}\cdot H_{\Delta z}\left(f_{x},f_{y}\right)\right\},n=1\\ F^{-1}\left\{F\left\{\sqrt{I_{n}\left(x,y\right)}⊕U_{n-1}\left(x,y\right)\right\}\cdot H_{\Delta z}\left(f_{x},f_{y}\right)\right\},1<n<N\\ F^{-1}\left\{F\left\{\sqrt{I_{N}\left(x,y\right)}⊕U_{N-1}\left(x,y\right)\right\}\cdot H_{-z_{N}}\left(f_{x},f_{y}\right)\right\},n=N \end{array}\right. \end{array}$$

The above iterative process results in a multi‐depth diffraction field that is free from the diffraction missing phenomenon. Subsequently, this field is fed into the designed FCN to generate a multi‐depth hologram with reconstructed scenes that also exhibit no diffraction missing phenomenon.

## 5 Experimental Results and Discussion

**Figure 5:**
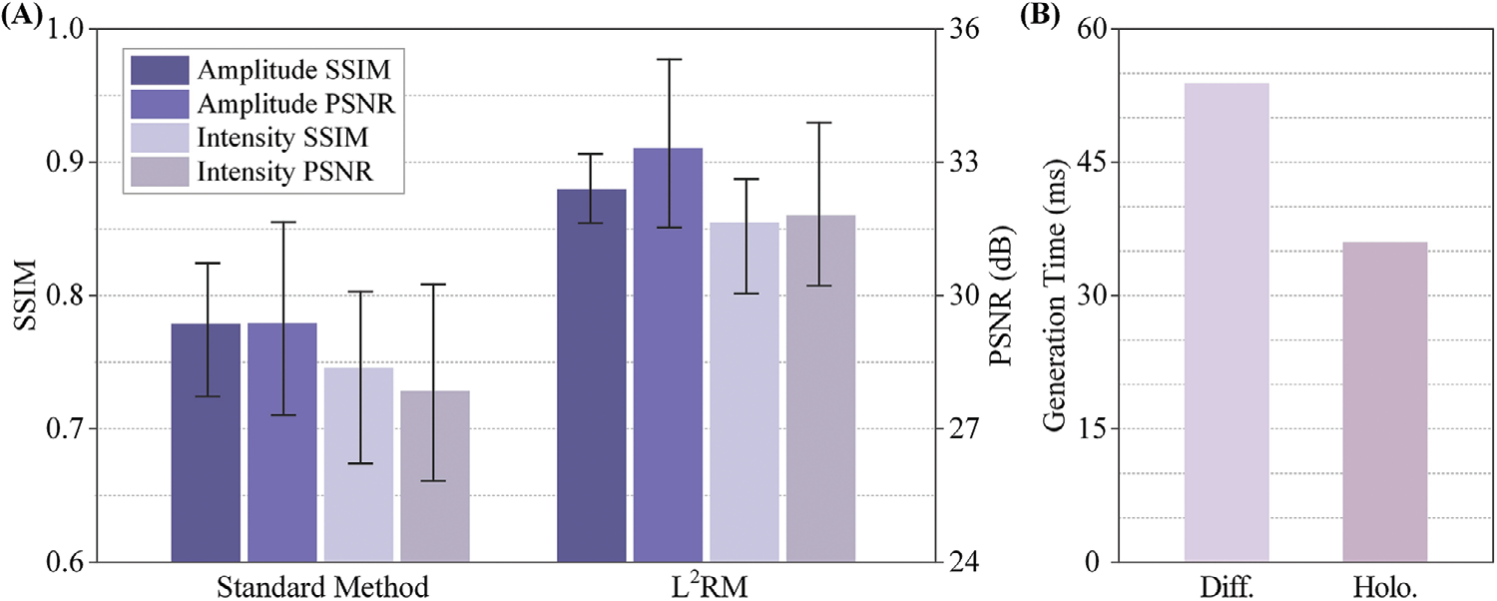
Evaluation for the generation of multi‐depth holograms. A) Reconstruction quality of holograms generated by the standard method and L^2^RM. B) Generation time of diffraction fields and holograms with L^2^RM.

Since the SLM used can only modulate monochromatic light, the grayscale values of the dataset were used for training and testing the neural model in the actual experiment. If the hardware equipment is sufficiently good, it is feasible to generate holograms separately for each color channel of the dataset and reconstruct a color image accordingly. Similar to 2D reconstructed images, we calculate the Peak Signal‐to‐Noise Ratio (PSNR) and the Structural Similarity Index (SSIM) by comparing the intensity image of the 3D scene with the recombination of the reconstructed diffraction fields. Since the forward–backward‐diffraction framework and FCN are fundamental methods employed in this paper, the standard method discussed in the experiments specifically pertains to “forward‐backward‐diffraction framework + FCN + standard method,” with L^2^RM denoting “forward–backward‐diffraction framework + FCN + L^2^RM.” We generated multi‐depth holograms of testing datasets and evaluated their reconstruction quality, as illustrated in **Figure** **5**A. The average intensity PSNR of the reconstructed scenes using L^2^RM is 31.8 dB, with an SSIM of 0.86, which is 4.0 dB and 0.11 higher compared to the standard method, respectively. As shown in Figure 5B, the average processing times for generating a multi‐depth diffraction field from a 3D scene and generating a multi‐depth hologram using FCN are 54 and 36 ms, respectively. Therefore, on a single GPU computing platform, it takes ≈90 ms to generate a multi‐depth hologram from a 3D scene. To achieve faster processing speeds, multiple GPUs can be employed to establish a parallel computing system where each GPU handles the computation of the diffraction field for a single layer (≈9 ms). Operating in a pipelined manner, this parallel computing setup can enhance the efficiency of large‐scale tasks, such as generating 3D holographic videos, theoretically approaching 36 ms per frame.

**Figure 6:**
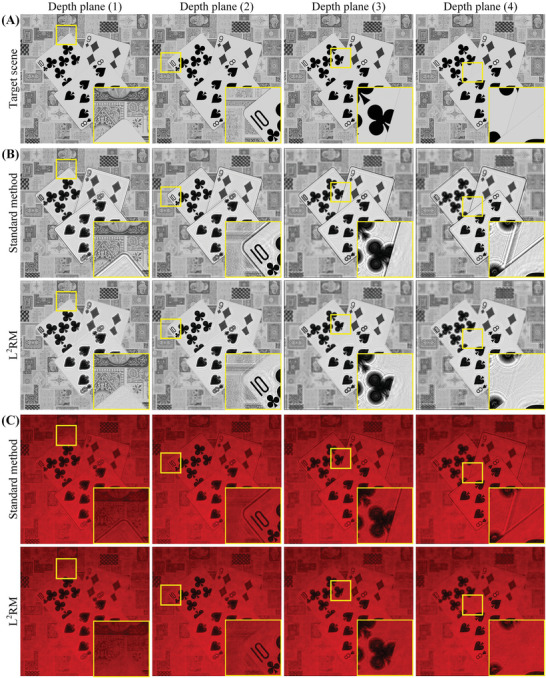
Quality comparison of reconstructions. A) Target scene. B) Numerical reconstruction of standard method and L^2^RM respectively. C) Optical reconstruction of standard method and L^2^RM respectively.

We reconstructed the “poker” scene shown in Figure 3B using both the standard method and the proposed L^2^RM, with the results presented in **Figure** **6**.

Figure 6B shows the numerical reconstructions of two methods in different layers. It is obvious that when implementing the occlusion culling in diffraction calculation, L^2^RM effectively alleviates the diffraction missing phenomenon existing in the standard method and enhances the reconstruction quality. As the counterpart of the numerical reconstruction, Figure 6C shows the optical experimental results for the standard method and the proposed L^2^RM. The experimental results give identical validation as that of the numerical ones, which indicates that the proposed L^2^RM can implement the occlusion culling well.

**Figure 7:**
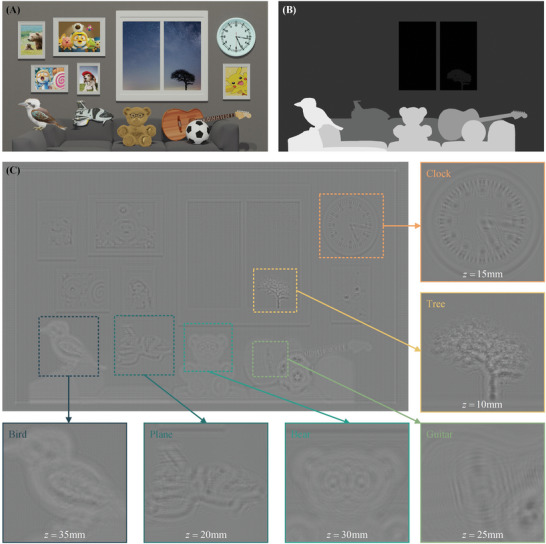
The complex 3D scene and the corresponding hologram. A) Intensity image and B) depth image of the 3D scene. C) The multi‐depth hologram generated by FCN.

In order to verify the effectiveness of the proposed method for complex scenes, we constructed a 3D scene depicting a living room and sampled it. The 3D scene is shown in **Figure** **7**A, and the main objects of interest are the tree, clock, airplane, guitar, bear, and bird. The corresponding depth information is divided into six sections, as depicted in Figure 7B. After calculating the multi‐depth diffraction field using the proposed L^2^RM and inputting it into the trained network model, we obtained the multi‐depth hologram of this 3D scene, as shown in Figure 7C. It can be observed that the phase of the generated hologram is smooth at all depths, indicating that the diffraction orders can be separated with a simple operation. Additionally, the smooth variation of phase is likely that the reconstructed scene will exhibit reduced speckle noise.

**Figure 8:**
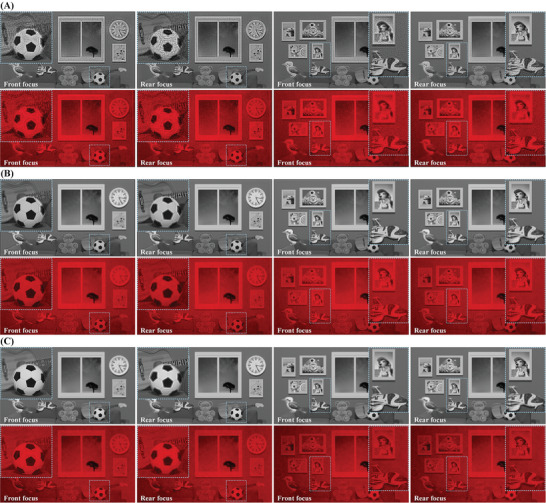
The numerical reconstruction and optical reconstruction of A) WH, B) DPH, and C) L^2^RM. The images in rows 1, 3, and 5 represent numerical reconstruction, while rows 2, 4, and 6 depict optical reconstruction. In columns 1 and 2, the camera focuses on the front focus plane (“football”) and the rear focus plane (“guitar”) of the “football‐guitar” pair, respectively. In columns 3 and 4, the camera focuses on the front focus plane (“airplane”) and the rear focus plane (“dog”) of the “airplane‐dog” pair, respectively.

To validate the effectiveness of L^2^RM, we choose WH^[^ ^16^ ^]^ based on iterative thinking and DPH^[^ ^17^, ^73^ ^]^ utilizing heuristic coding approximation as comparison techniques. The former involves 200 iterations, with a hologram generation speed of 6.5 s per frame and an average reconstructed image PSNR of 23.5 dB. The latter achieves a hologram generation speed of 60 ms per frame and an average reconstructed image PSNR of 28.6 dB. Numerical and optical reconstructions are independently generated for the complex scene illustrated in Figure 7 using WH, DPH, and L^2^RM. **Figure** **8** depicts these experimental outcomes. Our analysis focuses on the “football‐guitar” pair and “airplane‐dog” pair to contrast the defocus blur and image clarity among the reconstructions produced by the three methods. “Football” and “airplane” are positioned in the foreground, while “guitar” and “dog” are situated in the background.

Since each run of the WH algorithm optimizes for just one depth plane, 3D WH is composed of overlaying multiple 2D WH optimizations tailored to different depth planes. While the reconstruction quality of 2D WH is typically superior within the same group, this principle does not universally extend to 3D holography. In defocused scenarios, 2D WH exhibits a significant amount of speckle noise.^[^ ^16^ ^]^ Consequently, as shown in Figure 8A, the reconstructed images of 3D WH will display speckle noise proportional to the number of its depth planes. Furthermore, 3D WH also lacks support for smooth depth variations.

The principle of DPH involves representing any complex function as the sum of two unit complex functions. Just like the processing technique in 3D WH, we superimpose multiple depth planes of DPH to create a 3D DPH. A significant limitation of DPH is that the region with a high signal‐to‐noise ratio in the reconstruction plane is very narrow along the orientation of the DPH macropixels.^[^ ^74^ ^]^ Furthermore, the complex amplitude decomposition method of DPH diminishes the available spatial bandwidth product, resulting in the loss of high‐frequency details and causing the ringing effect (artifact) in the reconstructed image. Illustrated in Figure 8B, regions with abrupt grayscale changes like the transition between dark and light areas of a “football,” the edges of a “guitar,” and the tail of an “airplane” demonstrate prominent edge artifacts attributed to the absence of high‐frequency components.

The reconstructed image depicted in Figure 8C is generated using the method proposed in this paper. It is evident that, in comparison to WH, the reconstructed image from the proposed method exhibits minimal speckle noise. Similarly, when contrasted with DPH, there are also a few edge artifacts present in the reconstructed image. WH can only iteratively optimize the reconstructed image of a single depth plane, whereas FCN can simultaneously optimize the reconstructed images of multiple depth planes through a multi‐depth loss function. As a result, the proposed method can achieve seamless depth transformation without substantial speckle‐noise during defocusing. During the DPH generation process, two‐phase matrices, separated from the diffraction field, intersect using a “checkerboard” encoding method to create a phase matrix (phase‐only hologram).^[^ ^74^ ^]^ This decomposition and combination approach results in the loss of half of the phase information, leading to the high‐frequency components losing their carriers and consequently causing artifacts to appear in the reconstructed image. The primary method by which FCN processes diffraction fields is through convolution operations. While convolving the diffraction field, the dot product operation of the convolution kernel connects the real and imaginary parts of the diffraction field, while the sliding operation links neighboring elements within the diffraction field.^[^ ^75^ ^]^ After a finite number of convolutions and transposed convolutions,^[^ ^76^ ^]^ the receptive field of the generated hologram surpasses the diffraction range corresponding to the diffraction angle. We consider this as the foundation for neural networks to reconstruct diffraction fields. Expanding on this foundation, by training and optimizing FCN, it becomes feasible to retain high‐frequency components to a significant extent. Consequently, in comparison to DPH methods struggling to effectively retain high‐frequency components, the proposed method yields reconstructed images with minimal edge artifacts.

**Figure 9:**
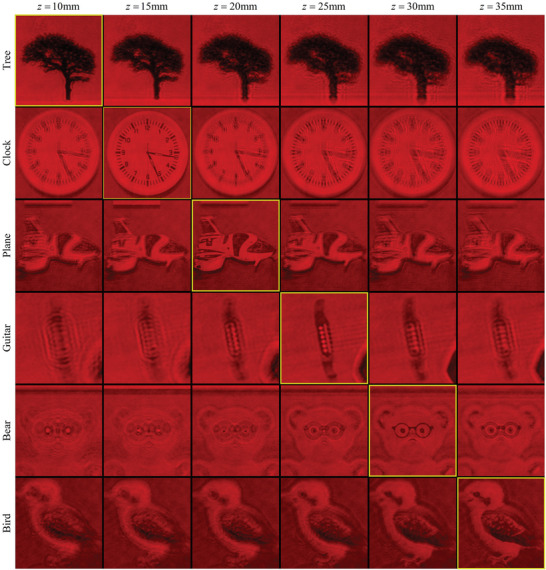
Reconstructed objects at different depth planes.

Furthermore, we respectively focus the camera on the depth planes of 10, 15, 20, 25, 30, and 35 mm. As depicted in **Figure** **9**, we capture the optical reconstructions of the sub‐objects at each depth, including the tree, clock, airplane, guitar, bear, and bird. It can be observed that all objects of interest exhibit clear reconstructions at their corresponding depths while appearing blurry at incorrect depths. Additionally, the optical reconstructions of the multi‐depth hologram show minimal speckle noise. Edge artifacts only manifest around objects that are out of focus, and they can be significantly reduced when properly focused.

## 6 Conclusion

In this paper, a random 3D training data set, a fully convolutional neural network and the multi‐depth loss function are introduced for generating phase‐only holograms from the multi‐depth diffraction field of a complex scene. Furthermore, the L^2^RM is introduced to implement the occlusion culling and to smoothen the boundaries between different layers during the reconstruction process. Numerical and optical experiments have demonstrated that the reconstructed scene exhibits high display quality and excellent 3D depth‐focusing.

In the next stage of our research, we plan to incrementally decrease the depth interval until reaching a certain level of continuous 3D reconstruction. However, when the depth interval is reduced, the depth‐focusing may not be apparent if no additional depth layers are added. But adding more depth layers may cause objects to be segmented improperly and lead to more boundary artifacts in the reconstructed image. Meanwhile, the large number of depth layers will greatly increase the computational cost of generating multiple‐depth diffraction fields. To address the aforementioned issues, we are considering introducing spherical waves and utilizing convolution methods to generate the diffraction field instead of ASM.

We also intend to achieve full‐color reconstruction of 3D scenes by introducing a three‐color laser through time‐division multiplexing. Certainly, aligning the diffraction patterns of different monochromatic light at the micron level is still a significant challenge. In addition, the generated model contains a large number of parameters, resulting in significant memory consumption and placing high demands on hardware devices, especially graphic processing units. On regular computing platforms, the generation speed of multi‐depth holograms will be significantly reduced, or even render the model unable to load due to insufficient graphics memory. Therefore, we plan to optimize the network model structure by reducing the number of model parameters, using lower‐precision parameters, compressing the model, and other methods to decrease memory usage and facilitate the fast generation of multi‐depth holograms.

## 7 Experimental Section

The numerical platform was based on Python 3.8.13, PyTorch version 1.11.0, and CUDA version 11.6. The designed FCN model was trained and tested on the NVIDIA Tesla A100 40G tensor core GPU. The Adam optimizer was used for optimizing the weights and biases with a learning rate of 0.0005. The model was trained for a total of 50 epochs.

**Figure 10:**
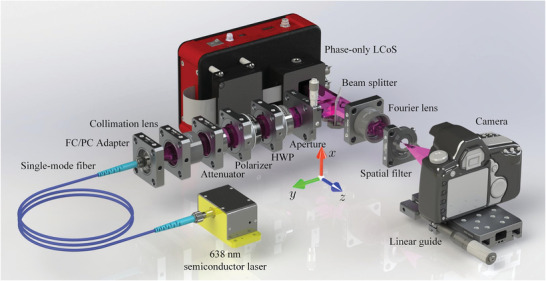
Optical experiment setup.

**Figure** **10** depicts the optical experiment setup where a non‐polarizing semiconductor laser with a wavelength of 638 (±8) nm, a power of 30 mW, and a single‐mode fiber with a core diameter of 4 µm were used as the reconstruction light source. The relatively broad linewidth could prevent the coherent noise so that the reconstruction quality can be improved. The output end of the fiber, which acted as a point source due to its small core diameter, was placed at the focal point of a collimated lens with a focal length of 100 mm to obtain plane waves. A neutral density filter was used as an attenuator and a polarizer to obtain linearly polarized light. The polarization orientation could be rotated with a half‐wave plate (HWP) so that it could be matched to the optimal polarization direction of the nematic twisted liquid crystal on silicon (LCoS) and a rectangular aperture was inserted to obtain a rectangular profile. The incident laser at the nematic twisted LCoS (Cas Microstar FSLM‐4K70‐P02) with a resolution of 4094 × 2400 and a pixel interval of 3.74 µm was modulated and reflected, and the reconstructed scene was further enlarged using a Fourier Lens with the focal length of 100 mm. A spatial filter was applied to allow the desired diffraction order to pass through while the other diffraction orders were filtered. The reconstructed magnified 3D scene was captured using a Canon EOS 5D Mark III camera, whose lens was removed and the scene was captured by the CMOS directly. The camera is positioned on a linear guide to adjust its location to capture the 3D scene at different depths.

### Statistical Analysis

The PSNR and the SSIM were introduced as the objective image quality function to evaluate the reconstruction of multi‐depth holograms. PSNR and SSIM are widely used metrics to measure the quality of a compressed or reconstructed image or video signal. Essentially, PSNR measures the similarity between two reconstructed scenes by comparing the peak signal magnitude and noise, while SSIM evaluates their structural information, such as luminance, contrast, and structure. They are respectively expressed as

(9) $$PSNR=10\cdot \log_{10}\left(\frac{1}{MSE}\right)$$

(10) $$SSIM=\frac{\left(2\mu_{e}\mu_{t}+c_{1}\right)\left(2\sigma_{e,t}+c_{2}\right)}{\left({\mu_{e}}^{2}+{\mu_{t}}^{2}+c_{1}\right)\left({\sigma_{e}}^{2}+{\sigma_{t}}^{2}+c_{2}\right)}$$

where *MSE* is the mean‐square error of the ground truth *I_t_* and the evaluated reconstruction *I_e_*, and $MSE=\frac{1}{MN}\sum {\left(I_{e}-I_{t}\right)}^{2}$. *M* × *N* is their size. $\mu_{e}$ and $\mu_{t}$ respectively represent their mean values. σ_*e*_^2^ and σ_*t*_^2^ respectively represent their variance, ${\sigma_{e}}^{2}=\frac{1}{MN-1}\sum {\left(I_{e}-\mu_{e}\right)}^{2}$ and ${\sigma_{t}}^{2}=\frac{1}{MN-1}\sum {\left(I_{t}-\mu_{t}\right)}^{2}$. σ_*e*,*t*_ is the covariance between them, $\sigma_{e,t}=\frac{1}{MN-1}\sum \left(I_{e}-\mu_{e}\right)\left(I_{t}-\mu_{t}\right)$. *c*_1_ and *c*_2_ are two constants, typically assigned values of 0.0001 and 0.0009, respectively. The higher the PSNR and SSIM values, the more similar the two scenes are, indicating superior reconstruction quality.

## Conflict of Interest

The authors declare no conflict of interest.

## Data Availability

The data that support the findings of this study are available from the corresponding author upon reasonable request.

## References

[1] Z. Luo , Y. Li , J. Semmen , Y. Rao , S.‐T. Wu , Light‐Sci. Appl. 2023, 12, 230. 37714841 10.1038/s41377-023-01254-8 PMC10504380

[2] H. Yoon , S.‐G. Oh , D. S. Kang , J. M. Park , S. J. Choi , K. Y. Suh , K. Char , H. H. Lee , Nat. Commun. 2011, 2, 455. 21878909 10.1038/ncomms1456

[3] R. Hirayama , D. M. Plasencia , N. Masuda , S. Subramanian , Nature 2019, 575, 320. 31723288 10.1038/s41586-019-1739-5

[4] D. Gabor , Nature 1948, 161, 777. 18860291 10.1038/161777a0

[5] L. Shi , B. Li , C. Kim , P. Kellnhofer , W. Matusik , Nature 2021, 591, 234. 33692557 10.1038/s41586-020-03152-0

[6] J. Geng , Adv. Opt. Photonics. 2013, 5, 456. 25530827 10.1364/AOP.5.000456 PMC4269274

[7] A. Maimone , A. Georgiou , J. S. Kollin , ACM T. Graphic. 2017, 36, 85.

[8] S.‐F. Lin , D. Wang , Q.‐H. Wang , E.‐S. Kim , Opt. Laser Eng. 2020, 126, 105895.

[9] D. Blinder , T. Birnbaum , T. Ito , T. Shimobaba , LAM 2022, 3, 572.

[10] P.‐A. Blanche , Light: Adv. Manufact. 2021, 2, 446.

[11] T. Sugie , T. Akamatsu , T. Nishitsuji , R. Hirayama , N. Masuda , H. Nakayama , Y. Ichihashi , A. Shiraki , M. Oikawa , N. Takada , Y. Endo , T. Kakue , T. Shimobaba , T. Ito , Nat. Electron. 2018, 1, 254.

[12] Y. Zhao , L. Cao , H. Zhang , D. Kong , G. Jin , Opt. Express. 2015, 23, 25440. 26480062 10.1364/OE.23.025440

[13] J. Zhang , N. Pégard , J. Zhong , H. Adesnik , L. Waller , Optica 2017, 4, 1306.

[14] X. Li , J. Liu , J. Jia , Y. Pan , Y. Wang , Opt. Express. 2013, 21, 20577. 24103930 10.1364/OE.21.020577

[15] D. Pi , J. Wang , J. Liu , J. Li , Y. Sun , Y. Yang , W. Zhao , Y. Wang , Opt. Lett. 2022, 47, 4379. 36048658 10.1364/OL.469463

[16] P. Chakravarthula , Y. Peng , J. Kollin , H. Fuchs , F. Heide , ACM T. Graphic. 2019, 38, 213.

[17] T. Shimobaba , A. Sawchuk , Appl. Opt. 1978, 17, 3874. 20208629 10.1364/AO.17.003874

[18] R. Gale , E. Schütz , P. Herzig , Appl. Opt. 1993, 32, 2526. 20820413 10.1364/AO.32.002526

[19] W. Frees , T. Kämpf , E. B. Kley , A. Tünnermann , Proc. SPIE 7927, SPIE, San Francisco, CA, USA, 2011.

[20] S. Tay , P.‐A. Blanche , R. Voorakaranam , A. V. Tunç , W. Lin , S. Rokutanda , T. Gu , D. Flores , P. Wang , G. Li , P. S. Hilaire , J. Thomas , R. A. Norwood , M. Yamamoto , N. Peyghambarian , Nature 2008, 2008, 451. 10.1038/nature06596 18256667

[21] P.‐A. Blanche , A. Bablumian , R. Voorakaranam , C. Christenson , W. Lin , T. Gu , D. Flores , P. Wang , W.‐Y. Hsieh , M. Kathaperumal , B. Rachwal , O. Siddiqui , J. Thomas , R. A. Norwood , M. Yamamoto , N. Peyghambarian , Nature 2010, 468, 80. 21048763 10.1038/nature09521

[22] G. Zheng , M. Kenney , H. Mühlenbernd , G. Li , T. Zentgraf , S. Zhang , Nat. Nanotechnol. 2015, 10, 308. 25705870 10.1038/nnano.2015.2

[23] L. Huang , X. Chen , H. Mühlenbernd , H. Zhang , S. Chen , B. Bai , Q. Tan , G. Jin , K.‐W. Cheah , C.‐W. Qiu , J. Li , T. Zentgraf , S. Zhang , Nat. Commun. 2013, 4, 2808.

[24] D. E. Smalley , Q. Y. J. Smithwick , V. M. Bove , J. Barabas , S. Jolly , Nature 2013, 498, 313. 23783627 10.1038/nature12217

[25] N. Savage , Nat. Photonics. 2009, 3, 170.

[26] Z. Zhu , Y. Wen , J. Li , Y. Chen , Z. Peng , J. Li , L. Zhu , Y. Wu , L. Zhou , L. Liu , L. Zong , S. Yu , Light‐Sci. Appl. 2023, 12, 151. 37331984 10.1038/s41377-023-01202-6 PMC10277279

[27] C. L. Panuski , I. Christen , M. Minkov , C. J. Brabec , S. Trajtenberg‐Mills , A. D. Griffiths , J. J. D. McKendry , G. L. Leake , D. J. Coleman , C. Tran , J. S. Louis , J. Mucci , C. Horvath , J. N. Westwood‐Bachman , S. F. Preble , M. D. Dawson , M. J. Strain , M. L. Fanto , D. R. Englund , Nat. Photonics. 2022, 16, 834.

[28] J. Dong , C. Jiang , S. Jia , Opt. Lett. 2016, 41, 4301. 27628382 10.1364/OL.41.004301

[29] M. Paturzo , V. Pagliarulo , V. Bianco , P. Memmolo , L. Miccio , F. Merola , P. Ferraro , Opt. Laser Eng. 2018, 104, 32.

[30] Z. Wan , Z. Wang , X. Yang , Y. Shen , X. Fu , Opt. Express. 2020, 28, 31043. 33115088 10.1364/OE.400587

[31] S. Ngcobo , I. Litvin , L. Burger , A. Forbes , Nat. Commun. 2013, 4, 2289. 23907358 10.1038/ncomms3289

[32] X. Lin , Y. Rivenson , N. T. Yardimci , M. Veli , Y. Luo , M. Jarrahi , A. Ozcan , Science 2018, 361, 1004. 30049787 10.1126/science.aat8084

[33] T. Zhou , X. Lin , J. Wu , Y. Chen , H. Xie , Y. Li , J. Fan , H. Wu , L. Fang , Q. Dai , Nat. Photonics. 2021, 15, 367.

[34] P. Georgi , Q. Wei , B. Sain , C. Schlickriede , Y. Wang , L. Huang , T. Zentgraf , Sci. Adv. 2021, 7, 9718. 10.1126/sciadv.abf9718 PMC8046362 33853788

[35] H. Zhou , X. Li , Z. Xu , X. Li , G. Geng , J. Li , Y. Wang , L. Huang , Photonics Res. 2022, 10, 678.

[36] H. Yang , P. He , K. Ou , Y. Hu , Y. Jiang , H. Jia , Z. Xie , X. Yuan , H. Duan , Light‐Sci. Appl. 2023, 12, 79. 36977672 10.1038/s41377-023-01125-2 PMC10050323

[37] Z. Shi , Z. Wan , Z. Zhan , K. Liu , Q. Liu , X. Fu , Nat. Commun. 2023, 14, 1869. 37015931 10.1038/s41467-023-37594-7 PMC10073211

[38] T. PWM , P. TC , Y. M. WU , Light‐Sci. Appl. 2018, 6, 10.

[39] D. Blinder , T. Nishitsuji , P. Schelkens , Image Process 2021, 30, 9418. 10.1109/TIP.2021.3125495 34757908

[40] T. Nishitsuji , D. Blinder , T. Kakue , T. Shimobaba , P. Schelkens , T. Ito , Opt. Express. 2021, 29, 12849. 33985032 10.1364/OE.421230

[41] Y.‐P. Zhang , F. Wang , T.‐C. Poon , S. Fan , W. Xu , Opt. Express. 2018, 26, 19206. 30114180 10.1364/OE.26.019206

[42] D. Yasuki , T. Shimobaba , M. Makowski , J. Suszek , M. Sypek , T. Kakue , T. Ito , Opt. Express. 2022, 30, 7821. 35299536 10.1364/OE.453541

[43] M. Sun , Y. Yuan , Y. Bi , S. Zhang , J. Zhu , W. Zhang , Opt. Express. 2020, 28, 34994. 33182955 10.1364/OE.410314

[44] C. D. Cameron , D. A. Pain , M. Stanley , C. W. Slinger , Proc. SPIE 2000, 4109, 129.

[45] S.‐C. Kim , E.‐S. Kim , Appl. Opt. 2008, 47, 55.

[46] H. Kang , T. Yamaguchi , H. Yoshikawa , Appl. Opt. 2008, 47, 44. 10.1364/ao.47.000d44 18594578

[47] H. G. Kim , Y. Man Ro , Opt. Express. 2017, 25, 30418. 29221071 10.1364/OE.25.030418

[48] T. Shimobaba , T. Ito , Opt. Express. 2017, 25, 77. 28085812 10.1364/OE.25.000077

[49] T. Shimobaba , J. Weng , T. Sakurai , N. Okada , T. Nishitsuji , N. Takada , A. Shiraki , N. Masuda , T. Ito , Comput. Phys. Commun. 2012, 183, 1124.

[50] H. Pang , J. Wang , A. Cao , M. Zhang , L. Shi , Q. Deng , IEEE Photonics J 2017, 9, 1.

[51] G. RW , W. O. Saxton , Optik 1972, 35, 237.

[52] Y. Wu , J. Wang , C. Chen , C.‐J. Liu , F.‐M. Jin , N. Chen , Opt. Express. 2021, 29, 1412. 33726357 10.1364/OE.413723

[53] G. Barbastathis , A. Ozcan , G. Situ , Optica 2019, 6, 921.

[54] Y. Rivenson , Y. Wu , A. Ozcan , Light‐Sci. Appl. 2019, 8, 85. 31645929 10.1038/s41377-019-0196-0 PMC6804620

[55] W. Ma , Z. Liu , Z. A. Kudyshev , A. Boltasseva , W. Cai , Y. Liu , Nat. Photonics. 2021, 15, 77.

[56] L. Shi , B. Li , W. Matusik , Light‐Sci. Appl. 2022, 11, 247. 35922407 10.1038/s41377-022-00894-6 PMC9349218

[57] G. Situ , Light: Adv. Manufact. 2022, 3, 278.

[58] S. Choi , M. Gopakumar , Y. Peng , J. Kim , G. Wetzstein , A. I. Claims , ACM T. Graphic. 2021, 40, 240.

[59] M. Eybpos , N. W. Cair , M. Atis , P. Chakravarthul , N. C. Pégard , Opt. Express. 2020, 28, 26636. 32906933 10.1364/OE.399624

[60] J. Lee , J. Jeong , J. Cho , D. Yoo , B. Lee , B. Lee , Opt. Express. 2020, 28, 27137. 32906972 10.1364/OE.402317

[61] D. Yang , W. Seo , H. Yu , S. I. Kim , B. Shin , C.‐K. Lee , S. Moon , J. An , J.‐Y. Hong , G. Sung , H.‐S. Lee , Nat. Commun. 2022, 13, 6012. 36224198 10.1038/s41467-022-33728-5 PMC9556550

[62] S. Sotthivirat , J. Fessler , J. Opt. Soc. Am. A. 2004, 21, 737. 10.1364/josaa.21.000737 15139426

[63] E. Williams , J. Acoust. Soc. Am. 2001, 110, 1976. 11681378 10.1121/1.1404381

[64] C. Chen , B. Lee , N.‐N. Li , M. Chae , D. Wang , Q.‐H. Wang , B. Lee , Opt. Express. 2021, 29, 15089. 33985216 10.1364/OE.425077

[65] P. Zhou , Y. Bi , M. Sun , H. Wang , F. Li , Y. Qi , Appl. Opt. 2014, 53, G206. 10.1364/AO.53.00G209 25322132

[66] Y. Ishii , F. Wang , H. Shiomi , T. Kakue , T. Ito , T. Shimobaba , Opt. Laser Eng. 2023, 170, 107758.

[67] G. Li , K.‐C. Kwon , G.‐H. Shin , J.‐S. Jeong , K.‐H. Yoo , N. Kim , J. Opt. Soc. Korea. 2012, 16, 381.

[68] K. Kim , H. Shim , Opt. Express. 2017, 25, 2666. 29519108 10.1364/OE.25.002666

[69] E. L. Francois , A. D. Griffiths , J. J. D. McKendry , H. Chen , D. D.‐U. Li , R. K. Henderson , J. Herrnsdorf , M. D. Dawson , M. J. Strain , Opt. Lett. 2021, 46, 3612. 34329237 10.1364/OL.424000

[70] K. Matsushima , T. Shimobaba , Opt. Express. 2009, 19662, 17. 19997186 10.1364/OE.17.019662

[71] X. Liu , X. Yan , X. Wang , Opt. Express. 2022, 30, 41624. 36366635 10.1364/OE.473205

[72] W. J. S. Goodman , Roberts and Company, Greenwood Village, John Wiley & Sons, Hoboken, NJ 2007.

[73] Y. Qi , C. Chang , J. Xia , Opt. Express. 2016, 24, 30368. 28059313 10.1364/OE.24.030368

[74] V. Arrizon , D. Sanchez‐de‐la‐Llave , Appl. Opt. 2002, 41, 3436. 12074515 10.1364/ao.41.003436

[75] A. Krizhevsky , I. Sutskever , G. Hinton , Communications of the ACM 2012, 60, 84.

[76] O. Ronneberger , P. P. Fischer , T. Brox , Arxiv abs/1505.04597 2015.

